# Costs of a successful public-private partnership for TB control in an urban setting in Nepal

**DOI:** 10.1186/1471-2458-7-84

**Published:** 2007-05-18

**Authors:** Deepak K Karki, Tolib N Mirzoev, Andrew T Green, James N Newell, Sushil C Baral

**Affiliations:** 1 Nuffield Centre for International Health and Development, Leeds Institute of Health Sciences and Public Health Research, University of Leeds, Leeds, LS2 9PL, UK; 2 Health Research and Social Development Forum, PO Box 24133, Kathmandu, Nepal

## Abstract

**Background:**

In South Asia a large number of patients seek treatment for TB from private practitioners (PPs), and there is increasing international interest in involving PPs in TB control. To evaluate the feasibility, effectiveness and costs of public-private partnerships (PPPs) for TB control, a PPP was developed in Lalitpur municipality, Nepal, where it is estimated that 50% of patients with TB are managed in the private sector. From the clinical perspective the PPP was shown to be effective. The aim of this paper is to assess and report on the costs involved in the PPP scheme.

**Methods:**

The approach to costing took a comprehensive view, with inclusion of costs not only incurred by health facilities but also social costs borne by patients and their escorts. Semi-structured questionnaires and guided interviews were used to collect start-up and recurrent costs for the scheme.

**Results:**

Overall costs for treating a TB patient under the PPP scheme averaged US$89.60. Start-up costs per patient represented 12% of the total budget. Half of recurrent costs were incurred by patients and their escorts, with institutional costs representing most of the rest. Female patients tended to spend more and patients referred from the private sector had the highest reported costs.

**Conclusion:**

Treating TB patients in the PPP scheme had a low additional cost, while doubling the case notification rate and maintaining a high success rate. Costs incurred by patients and their escorts were the largest contributors to the overall total. This suggests a focus for follow-up studies and for cost-minimisation strategies.

## Background

Tuberculosis is a leading cause of death worldwide [1] and, although the incidence rate is higher in many African countries, South Asia is the worst affected region in terms of absolute numbers [2]. The internationally recommended DOTS strategy [3,4] has been successfully implemented in the public sector by many National Tuberculosis Programmes (NTPs), but in the private sector the quality of care is generally very poor [5-7] This is a cause for concern since the private sector is a major provider of TB care in South Asia, particularly in urban centres [8]. There is currently considerable international interest in involving private practitioners (PPs) in TB control [9-11]. However, there is little documented evidence of the costs of such partnerships [12].

In Nepal, over 14,000 new cases of smear positive (i.e. infectious) tuberculosis are notified each year [13], and it has been estimated that in urban areas 50% of TB patients were (poorly) managed in the private sector [14,15]. Furthermore, with an average per capita income of US$378 [16], patients experience financial constraints to accessing TB treatment. Following consultation of various policy options, the Nepal NTP and the research team developed a public-private partnership (PPP) for TB control in urban Nepal. Lalitpur Municipality, a medium-sized city in the Kathmandu valley with a population of about 200,000 – a typical urban setting in the Nepalese context – was chosen as a pilot site for this initiative.

In Lalitpur Municipality, District Public Health Office (DPHO) is responsible for the TB control programme. The DPHO took responsibility for day-to-day management of the PPP scheme. This included providing training for laboratory staff and Directly Observed Treatment (DOT) supervisors, supervising workers involved in TB control, ensuring supply and distribution of medicines, and ensuring that five Treatment Centres (TCs) followed standard NTP recording and reporting guidelines.

A semi-governmental hospital (Patan Hospital), three NGOs (Yala Urban Health Programme (YUHP), Nepal Anti-TB Association (NATA) and Care & Fair) and one private nursing home (Hargan's Nursing Home, a private clinic running private outpatient clinics in the mornings and evenings [17]) were invited to become TCs for DOT.

An agreement was reached that microscopy services for the PPP would be provided by a laboratory at Hargan's Nursing Home, two laboratories run by NGOs, and a laboratory at Patan hospital. DOT would be provided by all five TCs. TCs had a memorandum of understanding with the DPHO; other agreements were informal.

PPs were asked to refer patients to the DOTS centres rather than providing DOT themselves; patients also self-referred. Although not specifically targeted under the PPP scheme, pharmacists could also refer suspected cases to the TCs. PPs in the scheme were seen as complementary to the public DOTS services, rather than replacing them, and were encouraged to use referral facilities for TB laboratory tests, DOT and complicated cases.

No financial incentives were paid to PPs, laboratories or TCs, and no other incentives were provided other than signs that confirmed participating DOTS centres were accredited by the NTP. Treatment centres paid their own staff's salaries. Patients paid the consultation fees when they visited private practitioners. As is normal in Nepal, the NTP covered costs of training, drugs for DOTS centres (but not for individual private practitioners), laboratory supplies (but not for private labs), and materials for recording and reporting. Full details of the organisation of the Lalitpur PPP are published elsewhere [17].

Two other groups were considered to be indirectly related to the PPP scheme: volunteers and traditional healers. The volunteers included in this study were unpaid local people, selected by Lalitpur municipality and responsible to the DOTS centre for community awareness, late patient tracing and visiting PPs and private pharmacies who referred TB cases to DOTS centres to deliver 'thank you' follow-up letters. Volunteers also provided a mechanism for the partnership to interact with PPs. They were provided training by YUHP in collaboration with the DPHO.

In this context traditional healers are local people who practice ethnomedicine and other alternatives to western medical practice and who are easily accessible by almost all households in Nepal. Traditional healers in Nepal are mainly faith-based and can be distinguished by the types of services (e.g. astrology and herbal medicine) they offer [18,19].

Clinical assessment demonstrated that the PPP met the international target for treatment success, and increased the number of TB patients receiving care under the DOTS strategy. The establishment of the PPP showed an overall increase of case notification of sputum positive patients in the study area from 54/100,000 before implementation in 1998 to 102/100,000 following implementation in 2001. Treatment success rates were over 90%, exceeding the international target of 85%, and less than 1% of patients defaulted. Details of the evaluation of the effectiveness of the PPP, and lessons learnt, are published elsewhere [20,21].

Although the PPP had been demonstrated to be clinically effective, it was considered important to assess its costs to allow others to assess feasibility of replicating the PPP scheme elsewhere [22]. This paper reports details of this study.

## Methods

The study used a comprehensive approach to describing costs, including costs incurred by DOT centres, patients and their escorts, under the PPP scheme. This enables assessment of the long-term sustainability of the PPP [23,24].

Semi-structured interviews were used to collect data on costs. Recurrent costs were collected during the financial year 2001–2002. All costs were collected in Nepali Rupees (NRs), which were converted into US$ at the 2002 exchange rate of 1US$ = 78 NRs [25].

The costs for conducting this study (research project costs) were treated as not directly related to the PPP and, although reported in this paper, were not included as part of costs for treating a TB patient.

All interviewees gave verbal informed consent prior to being interviewed. Ethical approval for this study was given by the Nepal Health Research Council.

Data on start-up and recurrent costs were collected in three major fields: *institutional costs*, including costs incurred by the DPHO and by each of five DOTS Centres or TCs; *patient-related costs *including charges, direct travel costs and opportunity costs for time lost in travel both by patients and their escorts; and *costs of involving volunteers *in the PPP.

**Figure 1:**
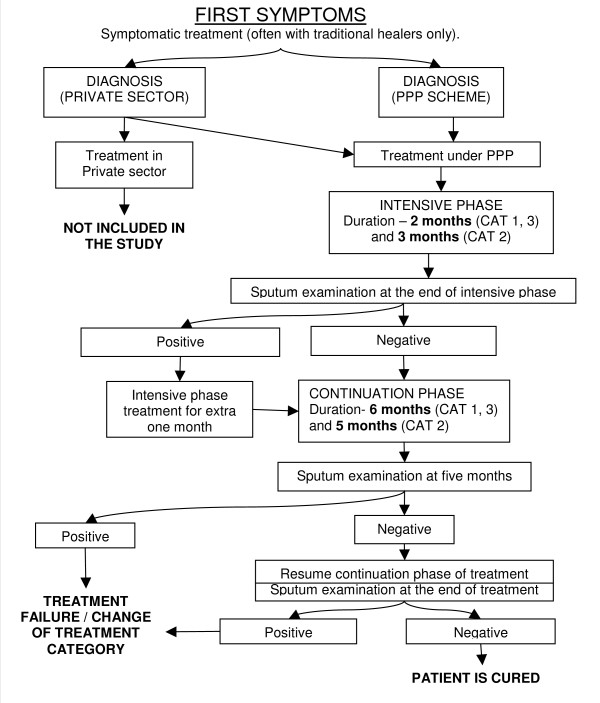
**Patient's treatment path**. Treatment paths of TB patients in Treatment Categories (CAT) 1, 2 and 3 are schematically presented. Only paths of patients involved in the PPP are shown. General treatment duration for all patients in CAT 1–3 is 8 months; if follow-up sputum examination at 2/3 months is found positive then patient gets treatment for 9 months. Diagnosis in the private sector was done by PPs who were encouraged to use referral facilities for TB laboratory tests under the PPP scheme, and patients were referred to the Treatment Centres for the complete course of treatment under the PPP scheme.

Start-up costs were estimated per patient and recurrent costs per patient per month. Costs were then calculated for treating a TB patient on average throughout the whole source of treatment. The average duration of treatment was assumed to be 8 months across all three standard treatment categories, although in practice this may vary slightly depending on the treatment category (Figure 1). This was done because the relatively small sample of patient-reported data did not allow accurate estimates of costs in each treatment category.

Table 1 shows the sample sizes and sampling duration used in the study.

**Table 1:** Sample size and sampling duration by field

Major fields of costs incurred	Sample size (No of patients)	Sampling duration in months (for recurrent costs)
**1. Institutional costs, including**		
**District Health Office**	509	12
**Treatment Centres, comprising**	509	12
***YUHP***	*191*	12
***Care & Fair***	*44*	12
***NATA***	*48*	12
***Patan Hospital***	*140*	12
***Hargan's NH***	*86*	12
**2. Volunteers **	503	6
**3. Patient-related costs**	50	7 (median)

Sample sizes and sampling durations are given for the institutions, volunteers and patients.

Two approaches for costing analysis were deployed, based respectively on means and medians of costs within the different fields. Although both median and mean values are reported in the paper, we emphasise the importance of medians of costs: in our view these are more representative and allow study results to be more usefully extrapolated to the rest of Nepal and to other similar contexts. However, mean values have also been reported to permit comparisons with other studies.

The following three sections provide the details of the data collection methodologies to assess costs incurred by institutions, volunteers and patients respectively.

### Institutional costs

The study assessed all start-up and recurrent institutional costs related to the PPP scheme, including training, supervision, staff salaries, costs of medicines, managerial set-up, logistical supplies, transportation and NTP central administrative costs.

DPHO start-up costs included the managerial set-up of the scheme as well as training of PPs, TC staff and volunteers. Administrative costs of training PPs and volunteers were included in DPHO start-up costs. Recurrent costs included costs of staff, procurement and delivery of medicines, refresher training courses and regular (4-monthly) DOTS workshops involving staff from the TCs. All five TCs worked independently and supervision and monitoring was done by the DPHO.

Training costs were covered primarily by the DPHO. Training costs reported under different TCs represent costs necessitated by staff cover during training.

Costs of medicines for each treatment category of patients were calculated based on standard Nepal NTP treatment guidelines for each of three treatment categories of TB patients. The costs of medicines reflect market prices in 2002/2003 and were estimated using government procurement information provided by the NTP. These costs were down-inflated by 2.9% – the official inflation rate for 2002 [26] – to obtain 2001/02 prices. Although in practice costs of medicines were covered by the Nepal NTP, in order to simplify presentation of results, these were regarded as part of the DPHO contribution. Costs of medicines exclude costs related to storage and transportation of medicines from national to local levels; these were included in other DPHO costs.

### Volunteers' costs

A total of 23 volunteers were interviewed to obtain estimates of their costs.

Whilst administrative costs of training volunteers and PPs were regarded as part of DPHO start-up costs, the opportunity costs for the time spent on training was included in volunteers' start-up costs. These were estimated as two days salary (480 NRs, US$6.20) for 43 people.

The recurrent expenses incurred by volunteers were recorded separately. These comprise costs reported for three major types of activities, namely tracing patients, visiting DOTS centres and meetings with PPs. Volunteers' costs include actual travel expenses as well as the opportunity costs of their time spent on the above activities.

### Patients' costs

Patients' costs were obtained by interviewing patients from each TC. Data were collected from patients registered in TCs in the period May 2002 to January 2003. Ten patients from each TC were randomly selected using the NTP TB register and TB treatment cards. Of the 50 patients interviewed, 27 were male and 23 were female; 26 patients were in treatment category I, 21 patients in category III and 3 in category II.

Patients' costs were collected across the following categories: costs incurred with traditional healers; charges during diagnosis and treatment, which included consultation and investigation charges with the latter comprising costs of sputum smear microscopy, X-ray examination, routine blood tests, Mantoux test and Lung Function Test (LFT); and travel costs including opportunity costs for patients and their escorts. Patients' costs include income losses due to TB illness during the period from when they first sought diagnosis from any source (including traditional healers) until the diagnosis of TB was confirmed.

No intervention was made to bring traditional healers into mainstream TB control; costs related to traditional healers represent those reported by patients. Patients' opportunity costs were estimated using *actual *income rates for each patient, with daily rates varying from US$1.60 to US$3.08.

## Results

Our estimates show that the median total cost involved in treating a TB patient in the PPP scheme is US$89.60 including start-up costs (Table 2). The mean cost of US$245.30 is 2.7 times higher than the median, largely due to the considerable skew in the distribution of patient-reported costs. As a result of this skew, we have focused on median costs, although mean costs are also reported for comparison purposes.

**Table 2:** Summary of start-up and recurrent costs (US$)

	**Institutional Costs**											
							
	**DPHO**		**All TCs**		**Total**		**Volunteer Costs**		**Patient Costs**		**TOTAL**	
*No of Patients*	*509*		*509*				*503*		*50*			
		
*Data collection period (months)*	*12*		*12*				*6*		7			

	Median	Mean	Median	Mean	Median	Mean	Median	Mean	Median	Mean	Median	Mean

**Start-up costs**												

Total reported costs	3,625		8,107		11,731		264.6					
Total costs per patient	**7.10**	7.10	**3.50**	15.90	**10.60**	23.00	**0.50**	0.50	**0**	0	**11.10 (12%)**	23.50 (9.6%)

**Recurrent costs**												

Total reported costs	10,404		11,673		22,077		508		7,952			
Total costs per patient per 8-month course	**20.80**	20.80	**14.80**	17.80	**35.60**	38.60	**7.00**	1.40	**35.90**	181.80	**78.50 (88%)**	221.80 (90.4%)

**TOTAL START-UP AND RECURRENT COST**	**27.9**	27.9	**18.3**	33.7	**46.2**	61.6	**7.5**	1.9	**35.9**	181.8	**89.60 (100%)**	245.30 (100%)

The breakdown of start-up and recurrent costs is presented with all costs being in US$.

Our estimate of the overall cost of treating a TB patient under the PPP scheme does not include research project costs (start-up research project costs were estimated as US$15 per patient and recurrent project costs were US$34 per patient per 8-month course).

The distribution of start-up and recurrent costs across institutions, volunteers and patients is given in Table 2 and in the following three sections.

### Start-up costs

Total start-up costs per patient were US$11.10, 64% of which were DPHO costs, 32% TC costs and about 5% volunteers' costs.

Start-up costs for both DPHO and TCs include four main categories: training, procurement of equipment, managerial set-up and social mobilisation (Table 3). About half (47%) of DPHO start-up expenditure was on equipment, the remainder comprising costs of training (32%) and managerial set-up (22%). Equipment costs also formed the majority (84%) of TCs costs, the remainder comprising of training (7%), and social mobilisation (7%) and managerial set-up (2%).

**Table 3:** Breakdown of institutional costs (all costs are in US$)

	**Institutional Costs**									
		
		**Treatment Centres**								
			
							**All TCs**		**TOTAL**	
							
	**DPHO**	YUHP	Care & Fair	NATA	Patan Hospital	Hargan's NH	Median	Mean	Median	Mean
*No of Patients*	*509*	*191*	*44*	*48*	*140*	*86*	*509*			
**Start-up costs**										
*Total reported:*										
Training	1,151	390	34	15	112	13		565		
Equipment	1,693	179	0	213	6,410	11		6,814		
Managerial set-up	780	86	9	1	63	0		159		
Social mobilisation	0	5	0	359	205	0		569		
**Total reported costs**	3,625	660	43	588	6,791	24		8,107		**11,731**
**Total costs per patient**	**7.10**	3.50	1.00	12.30	48.50	0.30	**3.50**	15.90	**10.60**	23.00
**Recurrent costs**										
*Total reported costs:*										
Staff	2,170	1,470	824	667	4,211	815		7,987		
Medicines	5,992	0	0	0	0	0		0		
Supplies and transportation	889	37	36	0	0	8		80		
Training	987	58	26	16	39	30		168		
Others	366	774	1,180	383	219	882		3,438		
**Total reported costs**	10,404	2,339	2,066	1,065	4,469	1,735		11,673		**22,077**
**Total costs per patient per 8-month course**	20.80	8.20	31.30	14.80	21.30	13.40	**14.80**	17.80	**35.60**	38.60

**TOTAL INSTITUTIONAL COSTS**	**27.90**						**18.30**	33.70	**46.20**	61.60

The breakdown of institutional start-up and recurrent costs is presented with all costs being in US$

Note 1: DPHO training costs include only training of TC staff related to the PPP.

Note 2: Total cost per patient across all Treatment Centres was calculated as the median of TC costs. For individual TCs, costs per patient are the mean start-up costs.

Note 3: Recurrent costs per patient per 8-month course across all Treatment Centres was calculated as the median of TC costs. For individual TCs, costs per patient are the mean of recurrent costs per patient.

### Recurrent costs

Recurrent costs show a more complex picture. As shown in Table 2, the distribution of overall costs for treating one TB patient comprised 40% for patient costs, 31% for DPHO costs, 20% for TC costs, and 8% for volunteers' costs.

#### Institutional recurrent costs

A breakdown of institutional recurrent costs is shown in Table 3. Costs incurred by the DPHO and TCs fall into four major categories: staff; procurement and transportation of medicines and (in the case of TCs) supplies; training-related (workshop) costs; and others including utilities, maintenance and miscellaneous expenses.

DPHO recurrent costs were made up of costs for procurement and distribution of medicines and supplies (70%), staff (20%) and training-related costs (10%). Central administrative costs of the Nepal NTP were estimated to be $7.20/patient treated and were added to the DPHO recurrent costs.

A breakdown of total reported TCs' recurrent costs shows that 38% of these costs were incurred by Patan hospital and that staff costs dominated (68% of total costs). The relatively low cost of supplies and transportation within TCs is due to the fact that procurement and transportation of medicines is covered by the DPHO. Care & Fair reported the highest costs of utilities and others among the TCs. These high costs are thought to be exceptional.

#### Volunteers' recurrent costs

As shown in Table 2, the costs per patient incurred by volunteers are relatively low. Table 4 provides a breakdown of this. Most volunteers' costs were incurred in attending monthly meetings at TCs, followed by patient tracing and regular visits to TCs, with a small fraction of costs incurred in meeting with PPs.

**Table 4:** Breakdown of total volunteers' recurrent costs (US$)

				**Cost per patient per 8-month course**	
				
**Functions of volunteers**	**Total reported cost**	**Min**	**Max**	**Median**	**Mean**
Tracing patients	**169**	**0**	**44.80**	**1.10**	0.40
Visiting the TC	**152**	**0.30**	**17.60**	**1.50**	0.40
Meeting PPs	**39**	**0**	**8.80**	**0.20**	0.10
Monthly meeting at the TC	**148**	**0.90**	**18.90**	**1.40**	0.40

**TOTAL VOLUNTEER COSTS**	**508**	**2.70**	**65.10**	**7.00**	1.30

Volunteer costs are presented across their main functions.

Note 1: Medians per patient per 8-month course are given for each volunteer activity separately; therefore the total in the medians column is not the sum of median costs.

#### Patients' recurrent costs

Costs reported by patients are broken down into 6 major categories reflecting the diagnosis and treatment periods. It is worth noting that medicines were provided to patients free of charge: these costs are part of the institutional recurrent costs. Costs of travel of both patients and their escorts have been differentiated from charges for diagnosis and treatment consultations.

Total reported patients' recurrent costs (Table 5) comprised patients' travel-related costs (78%) and charges incurred during diagnosis (13%). Other costs included costs with traditional healers, costs of escorts, treatment charges and other expenses.

**Table 5:** Patient-related recurrent costs (US$)

		**Costs**				
		
**Category of cost**	**Number of patients reporting non-zero cost**	**Total reported**	**Min value**	**Max value**	**Per patient per 8 months**	
					
					**Median**	**Mean**
Expenses with traditional healers	*11*	305	0	141	**0.0**	7.0
Diagnosis stage (excluding travel)						
*Consultation charges*	*48*	*196*	*0*	*16*	***1.5***	**4.5**
*Investigations*	*50*	*830*	*0.1*	*119*	***7.5***	*19.0*
**Sub-Total Diagnosis**		1,026	0.2	132	**10**	23.4
Treatment (excluding medicines and travel)						
*Consultation charges*	*14*	*66*	*0*	*15*	***0.0***	*1.5*
*Investigations*	*31*	*57*	*0*	*12*	***0.2***	*1.3*
**Sub-Total Treatment**		123	0	27	**0.3**	2.8
Patients' travel and opportunity costs						
*Travel expenses*	*11*	*184*	*0*	*37*	***0.0***	*4.2*
*Opportunity costs of days lost due to TB*	*18*	*5,152*	*0*	*1,755*	***0.0***	*117.8*
*Opportunity costs of travel time*	*50*	*842*	*1.8*	*202*	***8.8***	*19.3*
**Sub-Total Patients' Travel**		6,179	2.4	1,780	**3.8**	141.2
Costs of escorts (travel time lost + cost of travel)						
*Travel expenses*	*6*	*73*	*0*	*40*	***0.0***	*1.7*
*Opportunity costs of travel time*	*33*	*146*	*0*	*44*	***0.9***	*3.3*
**Sub-Total Costs of Escorts**		219	0	84	**0.9**	5.0
Other (miscellaneous) expenditures	*5*	100	0	36	**0.0**	2.3

**TOTAL PATIENTS' COSTS**		**7,952**			**35.90**	181.80

A breakdown of patient-related recurrent costs across main categories is presented. Frequency of costs from the total n = 50 is reported and total reported costs are shown versus median per patient's treatment course.

Note 1: The numbers of patients who reported costs are based on the total sample of 50.

Note 2: Minimum and maximum values and medians were derived for each type of costs separately and sub-totals in these columns are NOT the sums of their respective constituent costs.

Note 3: An increment from 7 months (median of patients' month at which costing data was collected) to 8 months (standard duration of treatment) was used to estimate mean values per patient per 8 months

Medians of costs incurred with traditional healers and other expenses were zero: that is, more than 50% of all patients did not report such costs. The largest median costs were for the diagnosis phase, and for opportunity costs of treatment-related travel.

Overall diagnosis-related costs were further divided into consultation charges (20%) and investigation-related charges (80%). (Some patients were charged for sputum lab tests, X-ray, and routine blood tests).

The majority of patients' non-medical costs comprised the opportunity costs for time lost due to travel to and from TCs, whilst financial transportation charges were reported in about 20% of cases and represent 18% of total patient-reported recurrent costs. The median cost of escorting patients was higher for female patients (US$1.83) than for male patients (US$0.11).

Treatment-related costs were a relatively small proportion of overall patient-related costs: this is to be expected as the NTP bears all the medical costs of treatment (for example, TB drugs and sputum examination in public health facilities). Though the median opportunity cost for the working time (rather than treatment time) lost by patients directly due to TB was zero in this study, for individuals these costs can represent a significant share of recurrent costs. For example, two patients reported not being able to work for 2 and 3 years: the corresponding maximum cost reported was US$1,755. Median patient and escort travel expenses were zero, reflecting the proximity of TCs to patients' homes/work places.

Patients' costs were also broken down by sex and source of referral (Table 6). The highest costs were incurred by patients referred from the private sector (US$56.30), followed by patients referred from semi-government facilities (US$44.50).

**Table 6:** Recurrent patient-related costs by sex and source of referral (US$)

**Source of referral**	**Male**			**Female**			**ALL (USD)**	
			
	**No of cases**	**Costs**		**No of cases**	**Costs**			
				
		**Median**	**Mean**		**Median**	**Mean**	**Median**	**Mean**
Public facility	13	**32.72**	128.44	10	**43.02**	99.00	**32.72**	115.64
Private sector	8	**30.63**	144.27	7	**68.35**	94.81	**56.31**	121.19
Self-referred	4	**63.74**	473.15	1	**32.94**	32.94	**30.17**	385.10
Semi government	2	**105.47**	160.42	5	**44.47**	472.84	**44.47**	383.58

**All referral sources**	**27**	**32.72**	186.57	**23**	**55.27**	176.13	**35.94**	181.77

A breakdown of patient-related recurrent costs by source of referral and sex is reported with No of cases indicating frequency of each. All costs are reported in US$).

Overall costs incurred by female patients were considerably higher than those reported by male patients. However, costs reported by male patients who were self-referred and referred from semi-government facilities were much higher than female patients, although the relatively small sample sizes within each category (for example, only two male patients were referred from semi-governmental facility and only one female patient was self-referred), may mean these findings are unrepresentative.

## Discussion

This study was designed to describe rather than compare costs. The costs of treating a TB patient within the PPP are estimated as US$89.60. The additional costs per patient to the public sector (start-up costs US$10.60 and recurrent US$35.60) associated with introduction of the scheme are small for the benefit of doubling the case notification rate. Two studies have been published that compare public sector costs and cost-effectiveness of TB control in PPP schemes [27,28], but none giving costs incurred within the routine Nepal NTP, so that we can not compare costs from our study with routine costs.

Several studies have estimated the mean costs of treating TB patients: in Pakistan estimated total costs of treating a TB patient varying from US$102 to US$180 [29]; in India total costs were estimated as US$120 [28], in Brazil the cost of treating one new TB case was about $103 [30], and in Ukraine the annual cost per TB patient was estimated as about US$124 [31]. In our study the total estimated cost of treating a TB patient calculated using medians (US$89.60) is generally lower than in studies reported in those studies whereas the cost calculated using mean values (US$ 239.90) is twice as high as most of these studies. This difference may largely be due to the different methodological approaches across the different studies – our study did not consider research project costs as part of the PPP and focused on median costs whereas the other studies used mean costs and mostly included project costs. Furthermore, none of the studies except the one in Salvador, Brazil considered patients' costs prior to diagnosis and start of TB treatment, whereas our study included income losses during the long delays in getting appropriate diagnosis and starting on treatment as part of patients' opportunity costs. Losses of income prior to diagnosis, although being significant for some patients, were reported only in a few cases and the use of medians helps to reduce the possibility of cost estimates being skewed.

Whilst mean values of total costs are from 2.6 to 3.8 higher than those estimated in Bangladesh, the median values of costs for treating one patient in our study are similar to those estimated in Bangladesh – slightly higher than in DOTS provided by BRAC (US$64) and lower than in the government DOTS programme (US$96) [32].

Likewise, NTP central level administrative costs in our study comprised 16% of the total public sector costs. This is comparable to the situation in Bangladesh [32] where public sector administrative costs including overheads represented 10% of the total public sector costs.

**Figure 2:**
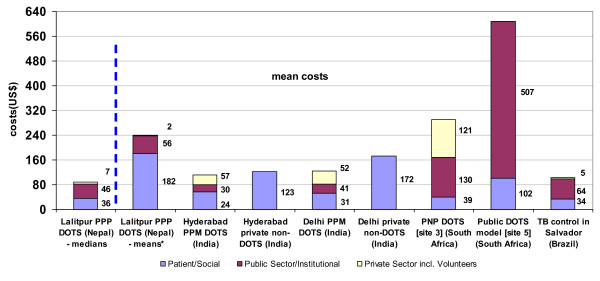
**Costs for treating a TB patient in Nepal, India, South Africa and Brazil**. Costs of treating a TB patient are presented in three broad areas: social or patient-related costs; public sector or institutional costs; and private sector costs. The study results were compared with costs of PPPs in similar studies in India [28], Brazil [30] and South Africa [27]. Note 1: Mean values of patients' costs were affected by the inclusion of costs prior to diagnosis and TB treatment. Note 2: costs incurred in the Nepal study include costs prior to diagnosis and treatment, in contrast to the other studies shown here. Note 3: care needs to be taken when comparing mean values, since these may be highly skewed

A major component of overall costs was patients' or social costs (Figure 2). Our finding that social costs constitute a significant share of the total cost for treating a TB patient was similar to the results from studies in India [28] and Brazil [30]. In Nepal, the NTP provides medicines free of charge, and these costs are regarded as public sector costs. Donation of time by volunteers in our study (see section 4.2) explains the relatively low private sector costs.

Several studies demonstrate that the DOTS strategy is more cost-effective than other approaches to TB control. As shown in Figure 2, a study in India has also shown that non-DOTS treatment of TB in the private sector is more expensive than DOTS implemented using public-private mix (PPM) [28]. Public-private partnerships in TB control nowadays are proving to be increasingly cost-effective, as demonstrated by studies in South Africa and India [27,28], which suggest that PPPs to deliver DOTS can remove a significant burden from the public health sector.

The dominance of recurrent costs over start-up costs mirrors the pattern generally seen when establishing a conventional DOTS programme. This pattern was also seen in Pakistan [29].

### Institutional costs

Analysis of start-up costs across different TCs re-affirmed a trend of larger facilities having generally higher institutional overheads. A high start-up institutional cost per patient was incurred by Patan Hospital (US$48.5) (Table 3). This is mainly explained by the construction of a TB clinic for DOTS, which cost US$45.79 per patient.

We have also found generally high start-up costs per patient incurred by one NGO. After a follow-up with the TCs we found that this largely due to their inability to distinguish between start-up costs that were attributed to the PPP scheme and other similar activities performed by the institution.

On the other hand, the relatively low start-up costs of Hargan's NH arise because no costs reported for managerial set-up and social mobilisation. This is explained by the previous focus of this private clinic on TB control, leading to a reduced need for start-up investments.

As for institutional recurrent costs, the DPHO and TCs incurred roughly the same total costs, indicating a need for strong financial commitment at all levels of the district health system. Staff costs and costs for procurement and distribution of medicines and supplies dominated within total institutional costs, representing respectively 46% and 32% of overall public recurrent costs.

Within TCs, the high recurrent costs per patient incurred by one of the NGOs (US$31.30) are largely explained by the relatively high cost of utilities (US$17.80 per patient), which, again, suggests an inability to draw a clear line between costs attributed to the PPP scheme and other institutional overheads.

All other TCs incurred recurrent costs in the range US$8.20 to US$21.30 per patient, which is considered reasonable, given the number of patients administered in each TC which varied from 48 to 191 and the size of the institution itself.

One of the messages which emerges from the above discussion is that high-cost institutions such as Patan Hospital (with high start-up costs) and Care and Fair (with high recurrent costs) may have a significant impact on the overall costs of the PPP scheme. This effect is likely to diminish if larger numbers of TCs are involved.

### Volunteers' costs

The costs of involving volunteers in the PPP scheme appear to be relatively low. Greater involvement of volunteers in the PPP scheme might be an interesting area to explore further as a potential for cost-minimisation strategies for the PPP scheme.

In this study we also assumed that volunteering had opportunity costs for patient tracing, meetings with PPs and visiting DOTS Centre, although in practice it was a voluntary donation of time in contrast to the involuntary time lost by TB patients. (Indeed interviews with volunteers indicate that it may be seen as an investment by individuals to gain subsequent employment opportunities). However, caution may be necessary to avoid overloading volunteers and to forestall volunteers' "compassion fatigue".

### Patients' costs

Patients' costs, including costs incurred by their escorts (in the form of direct charges for services and less obvious expenses such travel costs and time lost), represent the majority of estimated recurrent costs per patient and, perhaps, the most interesting area to explore in more detail. One emerging conclusion is that the significant financial burden on TB patients and their families may serve as a factor restricting patients from entering into, and completing, the full course of treatment. Further research is needed to confirm, further quantify and address this, and potential cost minimisation strategies should address, first of all, this aspect of recurrent budget costs.

#### Costs at diagnosis and treatment stages

A breakdown of patients' costs reveals a dominance of diagnosis-related charges over treatment-related charges. During treatment, consultation and investigation costs were similar, whereas during diagnosis investigation costs dominated.

Since the median month at which patients' costing data was collected was 7 months, i.e. towards the end of the average 8-months treatment course, we can be confident that most treatment-related charges were included in the study results. Clearly, most of the patients' expenditure occurred during diagnosis, whilst treatment-related charges represent a small fraction of the total patient expenses. The main conclusion emerging from this is that, from a costing perspective, attention should be given to cost-minimisation strategies during the diagnosis stage and in particular investigation-related costs as a strategy to reduce overall patient costs.

#### Travel-related costs

Transportation costs were reported in about 20% of cases. This relatively low frequency is normal for the urban context of our study. However, these, combined with the opportunity costs of time lost due to travel, represent a significant share of total costs within the study sample. This is primarily because TB treatment involves multiple visits to health facilities. If the decision is made to explore ways of decreasing patients' costs, this is seen as a potential area on which to focus attention.

An important feature of our study is the inclusion of patient opportunity costs due to time lost in travel which accounted for a substantial proportion of overall costs. We would therefore encourage other researchers to include the opportunity costs for economic days lost due to TB within social costs.

#### Costs of traditional healers

Another interesting finding is the relatively high costs incurred by patients who seek assistance from traditional healers. The frequency of these is relatively low (20%), but costs incurred for traditional healers reported by patients can be as high as US$141 (Table 5).

The traditional sector plays a significant role in determining patients' health in Nepal. Two potential strategies to address this issue are to encourage a move away from expensive healers providing ineffective treatment, or to equip these healers with basic knowledge of TB and clear instructions on referring potential TB cases.

#### Costs by gender

Treatment of female patients tends to cost more. This is probably because of cultural values in Nepali society, which require female patients to be escorted. Understanding this gender differences in seeking TB care is another area where research is needed.

Although this study was not designed to make statistical comparisons between costs for men and women, the cost differences between female and male patients represent an interesting area for a follow-up study.

The high patient-related costs seen in this study are likely to be similar for other TB control initiatives, whether Government or private. This suggests the need to address patient costs, including issues of equity and gender related to patient costs, more widely in TB control.

## Conclusion

This study has demonstrated that this PPP is a financially acceptable way of doubling TB case-finding while maintaining high success rates. There is, therefore, evidence to support scaling up this initiative in Nepal and in similar contexts. However, TB Programme managers will need to consider the degree to which PPs are willing to cooperate, as well as other contextual factors. Three particular issues are highlighted for consideration. Firstly, staff costs in the assessed PPP scheme are low compared to a number of other health systems. Secondly, an important feature of the initiative is the use of unpaid volunteers. Where this is not feasible, their roles may need to be replaced by paid staff, increasing total costs. Lastly, social (patient) costs estimated in this study, as in other TB control initiatives, represent a significant share of total costs, indicating a need to address issues of equity and gender to control costs to TB patients and their families.

## Competing interests

The author(s) declare that they have no competing interests.

## Authors' contributions

All authors contributed equally toward the preparation of this paper and are considered the first authors. More specifically, DKK participated in the study design, led the data collection, participated in analysis and preparation of the manuscript; TNM led the data analysis and coordinated the preparation of the manuscript; ATG coordinated the study, participated in data analysis and preparation of the manuscript; JNN conceived of the study, participated in the design of the study, advised on data collection and analysis and participated in the preparation of the manuscript; SCB participated in the design of the study, coordinated the data collection and participated in the data analysis and the preparation of the manuscript.

## Pre-publication history

The pre-publication history for this paper can be accessed here:


